# Characterization of Enterotoxigenic *Bacillus cereus* *sensu lato* and *Staphylococcus aureus* Isolates and Associated Enterotoxin Production Dynamics in Milk or Meat-Based Broth

**DOI:** 10.3390/toxins9070225

**Published:** 2017-07-15

**Authors:** Laura Walker-York-Moore, Sean C. Moore, Edward M. Fox

**Affiliations:** CSIRO Agriculture and Food, Werribee VIC 3030, Australia; lrwalkeryorkmoore@gmail.com (L.W.-Y.-M.); sean.moore@csiro.au (S.C.M.)

**Keywords:** *B. cereus s. l.*, *Staphylococcus aureus*, enterotoxin, milk, meat based broth

## Abstract

*Bacillus cereus sensu lato* species, as well as *Staphylococcus aureus*, are important pathogenic bacteria which can cause foodborne illness through the production of enterotoxins. This study characterised enterotoxin genes of these species and examined growth and enterotoxin production dynamics of isolates when grown in milk or meat-based broth. All *B. cereus s. l.* isolates harboured *nheA*, *hblA* and *entFM* toxin genes, with lower prevalence of *bceT* and *hlyII*. When grown at 16 °C, toxin production by individual *B. cereus s. l.* isolates varied depending on the food matrix; toxin was detected at cell densities below 5 log_10_(CFU/mL). At 16 °C no staphylococcal enterotoxin C (SEC) production was detected by *S. aureus* isolates, although low levels of SED production was noted. At 30 °C all *S. aureus* isolates produced detectable enterotoxin in the simulated meat matrix, whereas SEC production was significantly reduced in milk. Relative to *B. cereus s. l.* toxin production, *S. aureus* typically required reaching higher cell numbers to produce detectable levels of enterotoxin. Phylogenetic analysis of the *sec* and *sel* genes suggested population evolution which correlated with animal host adaptation, with subgroups of bovine isolates or caprine/ovine isolates noted, which were distinct from human isolates. Taken together, this study highlights the marked differences in the production of enterotoxins both associated with different growth matrices themselves, but also in the behaviour of individual strains when exposed to different food matrices.

## 1. Introduction

Foodborne diseases are diseases or illnesses that arise in an individual after they have ingested contaminated food or water. This contamination is frequently the result of contamination with pathogenic bacteria, some of which can cause illness through the production of enterotoxins [1]. These foodborne diseases often present with self-limiting gastroenteritis symptoms i.e., nausea, vomiting, diarrhoea, abdominal cramps, and fever, but severe complications can also arise, such as kidney and liver failure, brain and neural disorders, and paralysis, which can have fatal consequences.

The World Health Organization estimates the annual burden of foodborne illness to number 600 million cases of illness globally; of this, diarrheal illness accounts for the largest proportion of these cases and results in 230,000 deaths [1]. In Australia, it is estimated that there are 4.1 million cases of foodborne gastroenteritis per year, costing approximately AUS $1.2 billion [2,3]. These numbers, however, are likely underestimates due to a range of factors including non-mandatory reporting of cases to appropriate surveillance systems, lack of global surveillance systems, lack of appropriately-equipped testing facilities, and/or that those suffering mild symptoms may not seek medical attention [1,4,5]. The epidemiology of foodborne disease is important for the early detection of emerging trends. With the globalisation of food supply chains, it is important to not only have knowledge of the epidemiology of foodborne disease, but also of the causative agents implicated in these.

*Bacillus cereus sensu lato* species and *Staphylococcus aureus* are among the most important enterotoxigenic foodborne pathogens. These bacterial species produce a number of enterotoxins that have been implicated in multiple cases of foodborne disease, generally causing either emetic or diarrheal symptoms [6,7,8].

The *B. cereus s. l.* (or *B. cereus* group) is comprised of eight different Gram-positive species: *B. cereus sensu stricto*, *B. thuringiensis*, *B. weihenstephanensis*, *B. mycoides*, *B. pseudomycoides*, *B. cytotoxicus*, *B. toyonensis*, and *B. anthracis* [9,10,11]. They have the ability to express a number of enterotoxins including non-hemolytic enterotoxin (NHE), hemolysin BL (HBL), cytokine K (CytK), hemolysin II (HlyII), enterotoxin FM (EntFM), and enterotoxin T (bc-D-ENT) [12,13,14,15,16,17,18]. As spore-formers they can present an increased risk to food safety since spores may survive processing controls in food processing, such as pasteurization. 

*S. aureus* is a Gram-positive coccus and strains may possess a large repertoire of enterotoxins, as reviewed by Argudin et al. [19]. This includes over 23 heat-stable staphylococcal enterotoxins (SEs) including SEA to SEE, SEG to SEI, SEK to SET, and the staphylococcal enterotoxin-*like* proteins (SE*l*s) SE*l*J, and SE*l*U–SE*l*Y [20]. SEA to SEE are considered the ‘classical’ enterotoxins and have all been implicated in foodborne illness cases. SEH is the only non-classical enterotoxin that has also been implicated in causing disease outbreaks [21].

Gastroenteric infection caused by *B. cereus s. l.* species or *S. aureus* is thought to be almost exclusively through ingestion of contaminated foods [22]. To this end, understanding the behaviour of these pathogens in foods, including the dynamics of toxin production is vital to understanding the risks associated with contaminated foods. Data concerning what conditions contribute to the level of toxin production in foods is limited; in the case of *S. aureus,* most studies examined production in milk products [23,24,25]. A recent study found that for most isolates tested, both SED and SER were produced at higher levels in a meat food matrix relative to their production in milk [26]. Similar trends of lower enterotoxin production in milk were noted with *B. cereus s. l.* diarrheal toxins when compared with production in laboratory broth [27]. Another factor which is not fully understood is what number of bacterial cells are required to produce detectable levels of enterotoxin; some studies suggest 10^5^–10^6^ CFU per g or mL of food is generally required for *S. aureus* [28], and this rationale has formed the basis of food legislation [29]. In the case of *B. cereus s. l.* the infectious dose to elicit enterotoxigenic illness is again thought to be in the region of 10^5^–10^6^ cells, although investigations of contaminated foods which caused illness are sometimes at variance with this [30].

In this study enterotoxin gene profiles of bacterial species including *S. aureus* and four species of *B. cereus s. l.*, and associated production of enterotoxins in either milk or meat-based broth (MBB) food matrices were studied.

## 2. Results

### 2.1. Toxin Gene Screening of B. Cereus s. l. Isolates

**Figure 1:**
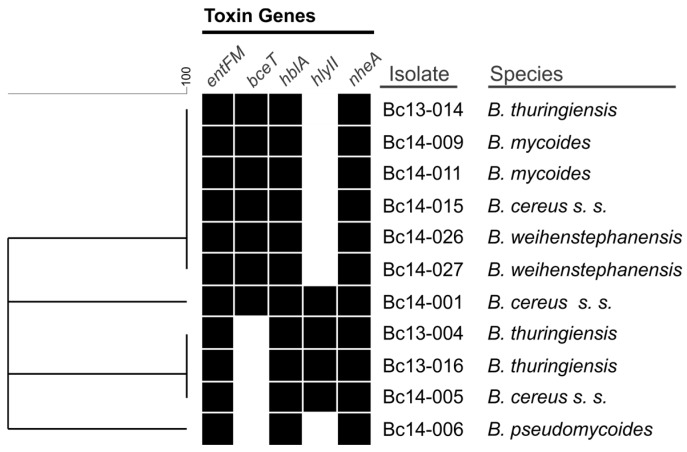
Similarity clustering analysis of the presence or absence of the five *B. cereus s. l.* enterotoxin gene targets. A black box indicates the presence of the gene, a white box indicates absence.

Isolates harboured at least three of the five toxin genes screened for. Only a single *B. cereus s. s.* isolate (Bc14-001) harboured all five of the toxin genes. All isolates harboured *entFM*, *hblA*, and *nheA* genes (Figure 1). It should be noted that Bc14-027 was negative for *hblA* by PCR detection, however, was positive based on the results of the lateral flow assay. The *bceT* gene was detected in six isolates, including *B. cereus s. s.*, *B. mycoides*, *B. thuringiensis*, and *B. weihenstephanensis* species. The *hlyII* gene was only identified in four isolates which belonged to either *B. cereus s. s.* or *B. thuringiensis* species.

### 2.2. Growth of B. cereus s. l. and Production of NHE and HBL in MBB or Milk Medium

**Figure 2:**
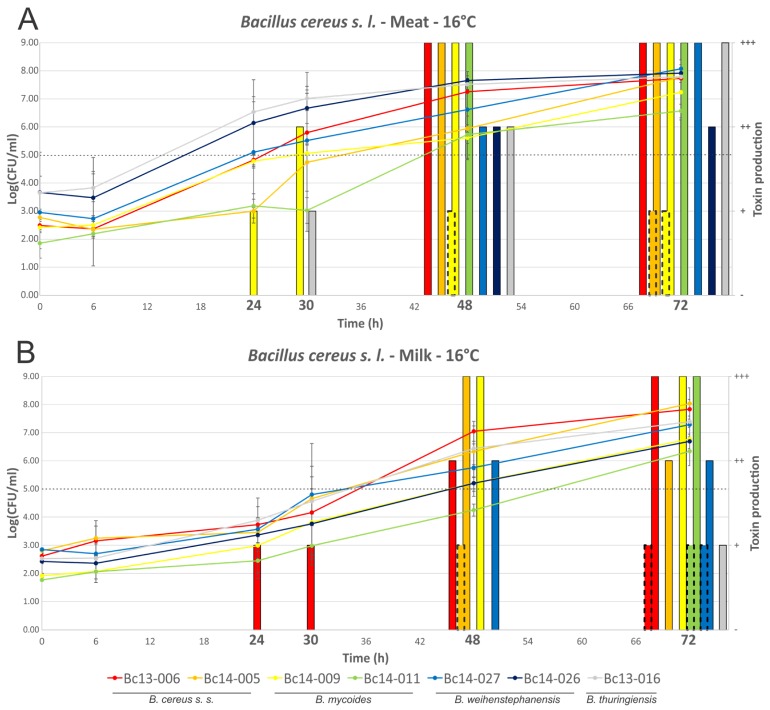
Growth dynamics and enterotoxin production of *B. cereus s. l.* isolates grown at 16 °C. Line graphs represent cell numbers and are plotted on the left *y*-axis. Quantified enterotoxin produced by isolates is shown by the bars and is plotted on the right *y*-axis. The continuous line bars represent NHE, with the broken border bars representing HBL. Time points are plotted on the x-axis, with those in bold indicating that enterotoxin detection was performed at that time point. (**A**) MBB medium; and (**B**) milk medium. The dotted line indicates the threshold which is thought to pose a significant food safety risk.

Growth and enterotoxin production (i.e., NHE and HBL) of seven *B. cereus s. l.* isolates is shown in Figure 2 (2A, meat; 2B, milk). Isolates grew to between 6.57 and 8.08 log_10_(CFU/mL) after 72 h in the meat-based medium, and to between 6.34 and 8.04 log_10_(CFU/mL) in the milk medium. Growth of isolates was not significantly different when comparing MBB to milk media, with the exception of isolate Bc14-026 which appeared to grow faster in MBB (significantly higher cell numbers noted at the 48 h timepoint in MBB relative to milk) and Bc13-016 (significantly higher cell numbers noted at timepoints 24 h and 30 h in MBB relative to milk). Production of NHE in the MBB was detected after 24 h (one isolate) and 30 h (two isolates), with all isolates producing NHE after 48 h and 72 h. All isolates produced intermediate (*n* = 1) or high (*n* = 6) levels of NHE in the MBB by 48 h, based on the semi-quantitative lateral flow device (LFD) detection method. In the milk medium NHE production was confirmed after 24 h (one isolate), 30 h (one isolate), 48 h (four isolates), and after 72 h (six isolates). One *B. weihenstephanensis* isolate (Bc14-026) did not produce detectable enterotoxin in milk at any of the time points tested; the other six isolates ranged from low to high NHE production by 72 h. HBL was only detected after 48 h in either broth (one isolate each); two or three isolates produced detectable levels of HBL after 72 h in meat or milk media, respectively. HBL was only produced at low levels when detected in either food matrix. Some differences in growth and toxin production were apparent when comparing the behaviour of a given isolate in each food matrix. Isolate Bc13-006, for example, produced detectable NHE after 24 h in milk, with a cell density of 3.73 ± 0.94 log_10_(CFU/mL). In contrast to this, no NHE production by this isolate was detected in MBB after 30 h, even though the cell density was 5.80 ± 0.33 log_10_(CFU/mL). After 48 h growth, however, the isolate produced high levels of NHE, with an associated cell density of 7.25 log_10_(CFU/mL). In the case of HBL production, although levels were low when produced, only Bc14-005 produced detectable HBL in both MBB and milk media under the conditions tested. Other isolates only produced detectable HBL in MBB (Bc14-009) or milk (Bc13-006, Bc14-01, and Bc14-027). Neither Bc13-016 nor Bc14-026 produced detectable HBL in either food matrix, despite harbouring associated toxin genes.

### 2.3. Growth of S. aureus and Production of SEC or SED Toxin in MBB or Milk Medium

**Figure 3:**
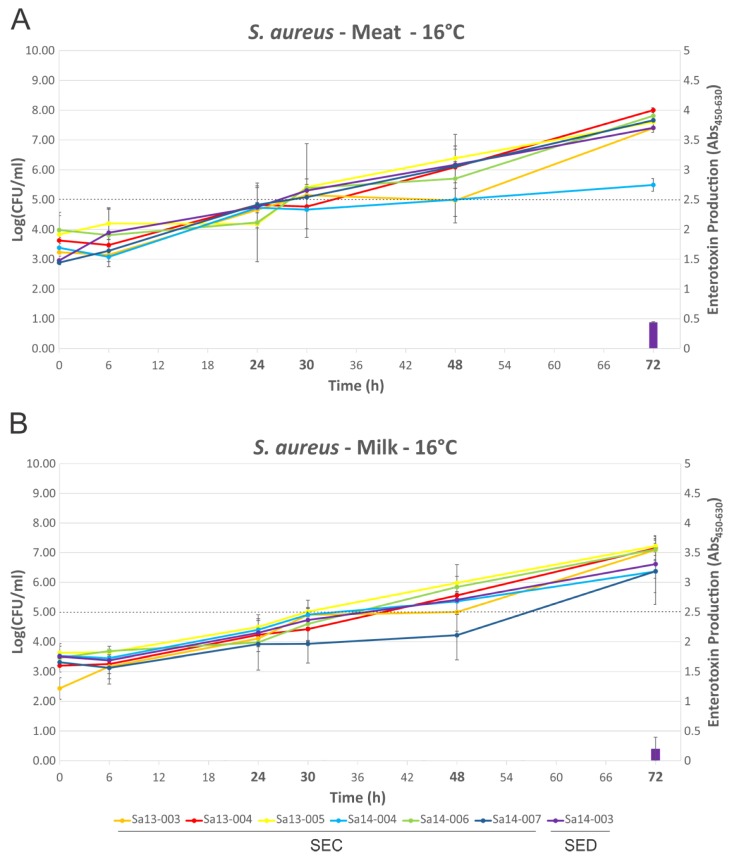
Growth dynamics and enterotoxin production of *S. aureus* isolates grown at 16 °C. Line graphs represent cell numbers and are plotted on the left y-axis. Enterotoxin production is shown by the bars and is plotted on the right y-axis. Time points are plotted on the x-axis, with those in bold indicating that enterotoxin detection was performed at that time point. (**A**) MBB medium; and (**B**) milk medium. The dotted line indicates the threshold which is thought to pose a significant food safety risk.

**Figure 4:**
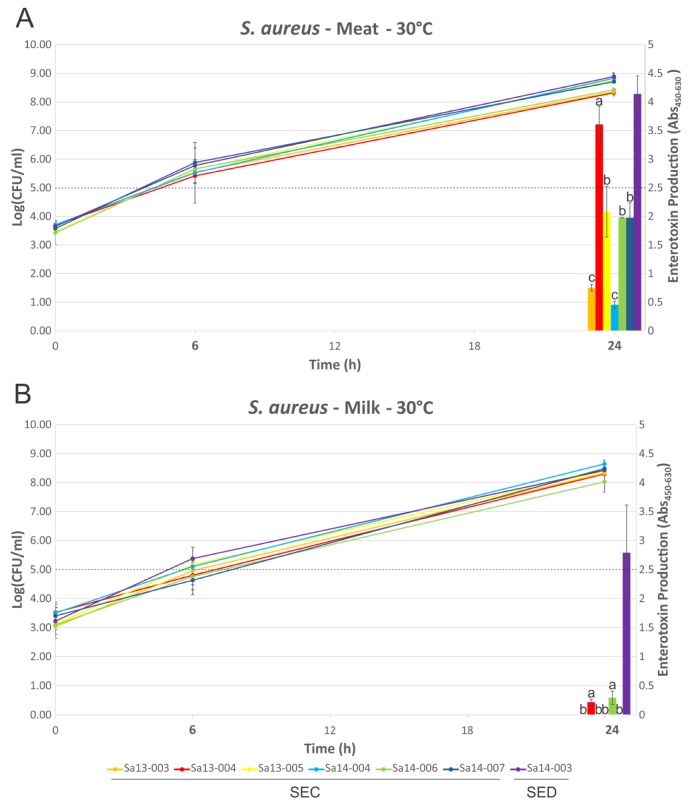
Growth dynamics and enterotoxin production of *S. aureus* isolates grown at 30 °C. Line graphs represent cell numbers and are plotted on the left y-axis. Enterotoxin production is shown by the bars and is plotted on the right y-axis. Time points are plotted on the x-axis, with those in bold indicating that enterotoxin detection was performed at that time point. (**A**) MBB medium; and (**B**) milk medium. The dotted line indicates the threshold which is thought to pose a significant food safety risk. Bars sharing a letter are not significantly different, whereas those with different levels were calculated as significantly different.

Growth of isolates followed a similar trend in MBB or milk medium when compared at 16 °C or 30 °C (Figure 3 and Figure 4), with few statistically significant differences identified (Sa13-005 and Sa14-006 have significantly higher cell densities after 72 h growth in MBB at 16 °C, *p* < 0.05). Isolates grew to between log_10_ 5.69 and 8.00 CFU/mL in MBB at 16 °C after 72 h, or between 6.36 and 7.22 log_10_(CFU/mL) in the case of milk under the same conditions. At 30 °C all isolates grew to between 8.32 and 8.89 log_10_(CFU/mL) or between 8.03 and 8.64 log_10_(CFU/mL) in MBB or milk, respectively. None of the isolates harbouring the *sec* gene produced detectable SEC at 16 °C up to 72 h growth, regardless of food medium. Sa14-003, which carried *sed*, only produced detectable toxin at the 72 h time point; low levels of SED production were noted in both MBB and milk medium, however, there was no significant difference in this production with respect to the media used. At 30 °C, however, greater differences in enterotoxin production were noted when comparing growth media. All isolates produced detectable enterotoxin (SEC or SED) after 24 h growth in the meat-based medium. Sa14-003 again showed no significant difference in production of SED, regardless of the medium it was grown in (*p* > 0.05). Of the SEC-producing isolates, the ovine isolate Sa13-004 produced significantly more enterotoxin after 24 h, relative to the other isolates. The lowest SEC enterotoxin production was observed for Sa13-003 (caprine) and Sa14-004 (bovine) isolates. Relative to SEC production in meat-based medium, all isolates produced significantly less enterotoxin in milk medium (Table 1). Only two isolates produced detectable SEC in milk at 24 h: Sa13-004 (ovine) and Sa14-006 (caprine).

### 2.4. Phylogenetic Analysis of sec and sel Toxin Genes

**Figure 5:**
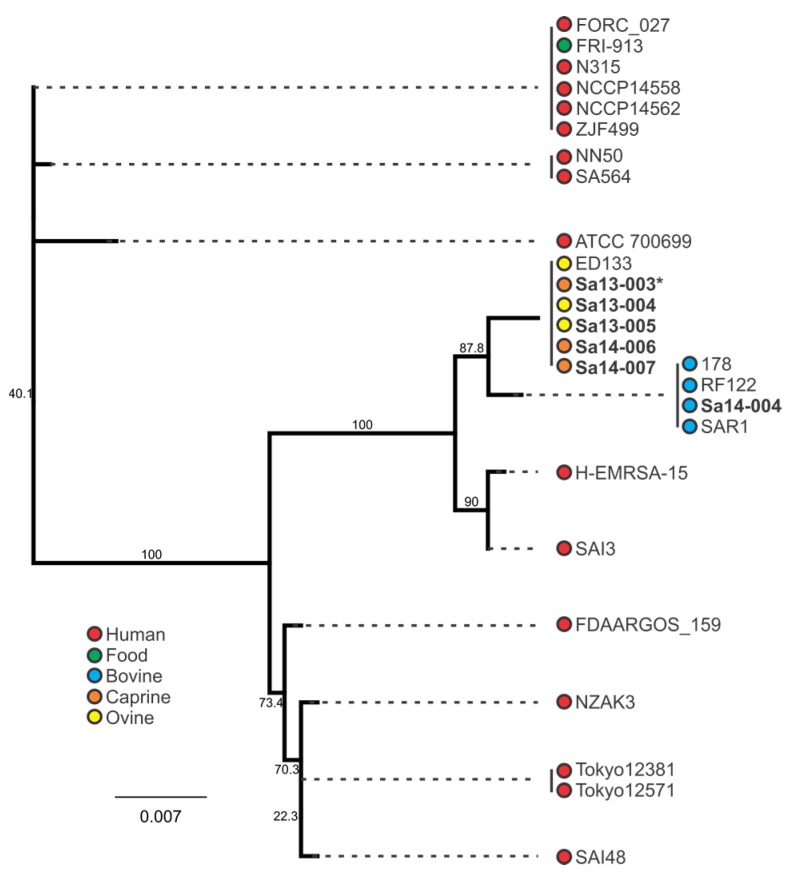
Phylogenetic analysis of *sec*-containing *S. aureus* isolates, with bootstrap values indicated. Isolates in bold were included in the growth profiling and enterotoxin production experiments, whereas those in normal font were obtained from the NCBI database. Source and colour associations are indicated in the legend. Isolates with a single deletion in an *sec* poly(A) homopolymeric tract are marked with an asterisk.

**Figure 6:**
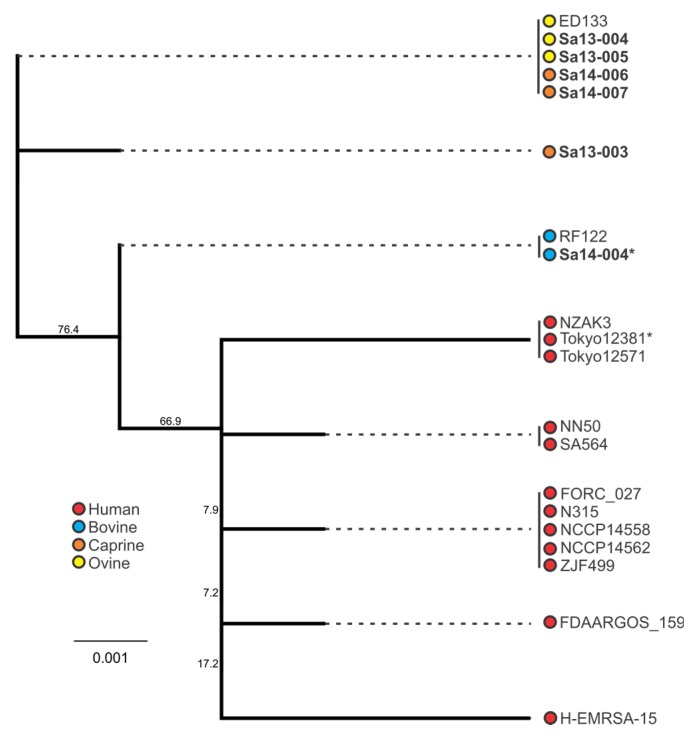
Phylogenetic analysis of *sel*-containing *S. aureus* isolates, with bootstrap values indicated. Isolates in bold were included in the growth profiling and enterotoxin production experiments, whereas those in normal font were obtained from the NCBI database. Source and colour associations are indicated in the legend. Isolates with a single deletion in an *sel* poly(A) homopolymeric tract are marked with an asterisk.

Phylogenetic clustering of the *sec* and *sel* genes are shown in Figure 5 and Figure 6, respectively. Inspection of the *sec* nucleotide sequence revealed that five of the six *sec*-carrying isolates encoded an 801 bp nucleotide gene sequence, with the remaining isolate (Sa13-003) having a single nucleotide deletion in its *sec* gene sequence. This deletion occurred in the poly(A) homopolymeric tract that consisted of a series of eight adenine nucleotides from positions 589-596 in the *sec* gene, resulting in only seven adenine residues. All caprine and ovine isolates clustered together when comparing *sec* nucleotide sequence, while a separate distinct group contained all bovine isolates (Figure 5). A clear segregation of animal and human isolates was also observed.

Similar to the trends observed with *sec*, the *sel*-harbouring isolates of animal origin clustered separately from human isolates (Figure 6). All caprine and ovine isolates formed two *sel* genotypes, with bovine isolates forming a separate cluster. Interestingly as with the *sec* gene, analysis of the *sel* nucleotide sequence of isolates in this study identified a single nucleotide deletion in the Sa14-004 bovine isolate; this deletion was also in a poly(A) homopolymeric tract, this one comprising seven adenine nucleotide residues in the wild-type gene sequence. Both *sec* and *sel* deletions resulted in premature stop codons.

## 3. Discussion

Diarrheal illness through consumption of contaminated foods remains an important issue for food safety and public health. Legislation is set to help ensure food products do not contain levels of pathogenic organisms which could cause illness if consumed, however, these often do not account for the behaviour of pathogens in specific food matrices due to a lack of related scientific data. Previous studies have examined aspects relating to the behaviour of the toxigenic species of *B. cereus s. l.* or *S. aureus*, including addressing considerations such as cell numbers, growth medium, temperature or specific enterotoxin. However although providing insights, these studies are often limited in that they rarely consider the combined impact of all these conditions, with particular context referenced to real life conditions relevant to current food chain paradigms. Understanding toxin production together with cell density under temperature abuse conditions is critical to informing food safety. To this end, previous studies may be limited as a result of using laboratory broth rather than a food matrix [31], not providing adequate consideration for cell density [26,27], only examining relatively high temperatures [25], or not considering variable behaviour in different food matrices [32]. With the globalization of food supply chains, increasingly complex logistics presents an increasing opportunity for temperature abuse. This study examined characterized the toxin genes of two important foodborne pathogens, *B. cereus s. l.* and *S. aureus*, and examined the dynamics of enterotoxin production in MBB or milk food matrices.

### 3.1. Toxin Gene Carriage among B. cereus s. l. Isolates in this Study

A key feature of the *B. cereus s. l.* family of bacteria is their wide repertoire of toxin genes [30]. NHE and HBL are three component pore-forming cytotoxic enterotoxins and among the most important of *B. cereus s. l.* toxins in the diarrheal form of illness, and were present in all 11 isolates screened in this study. Although *hblA* was not detected in Bc14-027 by the PCR assay, production of HBL by this strain was detected using the lateral flow device. This result underlies the limitations in using PCR detection to evaluate the presence of enterotoxin genes in *B. cereus s. l.*; these genes are known to be highly polymorphic, leading to false negatives using PCR screening due to variations in the primer target nucleotide sequence [33]. Evidence suggests that, of these enterotoxins, NHE contributes more significantly to diarrheal illness, although it is likely that secretion of multiple enterotoxins amplifies the severity of illness [34,35]. This is further supported by the observation that clinical isolates frequently contain a higher number of key enterotoxin genes relative to non-clinical isolates, with higher relative expression of enterotoxins noted in clinical strains [33]. The high rate of carriage NHE and HBL genes among isolates in this study suggests a wider assessment of *B. cereus s. l.* isolates linked to food production systems in Australia would be of value for risk assessment. Although HlyII shows both haemolytic and cytoxicity to human cells [17,36], it has not been identified as the etiological factor in diarrheal illness. Indeed, it is not regulated by the main virulence regulator PlcR, but rather is likely regulated by the iron uptake system regulator Fur [30,37]. This suggests that this secreted haemolysin is involved in iron scavenging. The *hlyII* gene was identified in four of the 11 isolates; this may provide a competitive advantage to these isolates over their *hlyII*-negative counterparts in iron-limited environments, however a more comprehensive study on iron uptake systems in these isolates is required to evaluate this. Evidence suggests that the enterotoxins BceT and EntFM also contribute to the severity of diarrheal illness [16,38]. With all isolates containing *bceT* and 64% also harbouring *entFM*, this may contribute to the severity of diarrheal illness induced by these isolates. This prevalence of *entFM* among *B. cereus s. l.* isolated from dairy farms was shared by a previous study from Ghana [39] and another from Brazil [40]; interestingly, the latter study found a higher carriage rate of *bceT* food poisoning strains relative to those isolated from foods or soil, although the majority of isolates among all groups carried this enterotoxin gene. The carriage of *entFM* among isolates in this study appears higher than previously reported in others [39,41], although a larger number of isolates would provide greater insight into the distribution of this enterotoxin among strains in Australia.

### 3.2. Behaviour of B. cereus s. l. in Food Matrices

Distinct differences in enterotoxin production were noted when comparing the behaviour of individual isolates in MBB versus milk media at 16 °C, a temperature reflecting food chain abuse conditions (Figure 2). Of particular interest was the observation that higher enterotoxin production was not associated with the same medium, but rather varied depending on the isolate. While Bc14-009 produced NHE earlier in MBB compared with milk, the opposite was observed with Bc13-006. This could not be strictly correlated to higher cell densities as most isolates did not show significant differences in growth dynamics. Although Bc13-016 grew to high cell numbers faster in MBB relative to milk with respect to 24 h and 30 h growth, and higher associated NHE production was noted at these time points, the higher NHE produced after 72 h growth by this isolate did not correlate with significantly higher cell density. Bc14-026 had higher cell numbers and NHE production after 30 h growth in MBB relative to milk. Although this isolate did not produce detectable NHE in milk, it should be noted that NHE production in meat was associated with cell densities higher than that achieved by this isolate in the milk medium. In all other cases the medium itself (or its associated characteristics), and not higher cell density, correlated to increased enterotoxin production. Bc13-006 produced detectable NHE after 24 h at an average cell density of 3.73 log_10_(CFU/mL), however, in MBB production of NHE was only detected after 48 h growth with an associated cell density of 7.25 log_10_(CFU/mL). This highlights the limitations of arbitrary tolerance limits in foods, and moreover emphasises the constraints of designating a strain as a low or high toxin producer based on limited testing which does not consider a broad range of food matrices. That withstanding, current opinion suggesting a tolerable limit below 10^3^–10^5^ CFU/g is generally supported by the results of this study with respect to diarrheal illness [42].

The current paradigm for *B. cereus s. l.* diarrheal illness suggests that toxin is produced during outgrowth of the bacterium in the gut, with toxin in foods inactivated in the stomach and, thus, not reaching the intestine in an active state [42,43]. However there are notable assertions underlying this premise, and findings from a number of studies which should be considered on this subject. Studies including gastric survival assays, which form the basis of the diarrheal illness paradigm, often omit the inclusion of food matrices [44]. Previous work has shown that inactivation of enteric pathogens in low pH broth assays do not reflect gastric simulated conditions where a food matrix is used [45]. Food particles can provide a near-neutral pH zone for other particles [46], which could include bacterial cells or toxins; indeed gastric pH during meal consumption can raise to close to neutral levels before it begins to decrease again [47]. In addition to this, stomach pH is known to vary with age, with elderly people for example typically having a higher stomach pH [46]. With evidence for greater stability of vegetative cells and toxins at higher gastric pH (particularly with regard to the associated food matrix), couple with the movement of chyme out of the stomach through gastric emptying before the pH returns to a low state suggests high levels of vegetative cells and/or toxins in foods may result in transit through the stomach without inactivation [48,49,50]. While there is a number of studies supporting the survival of vegetative cells, further studies with improved modelling of gastric transit with different food matrices coupled with cytoxicity testing are required to further understand these dynamics [51].

### 3.3. Behaviour of *S. aureus* in Food Matrices

Although growth was largely comparable in both food matrices regardless of temperature, it is clear that the strain and/or food matrix can lead to substantial differences in enterotoxin production (Figure 3 and Figure 4). Previous studies have suggested that growth phase may influence enterotoxin gene expression and/or production, although the specific growth phase(s) associated with maximal expression/production may themselves be variable depending on the enterotoxin in question [23,25]. At 16 °C no SEC production by any isolate was detected, suggesting that temperature abuse of milk or beef at, or below, this temperature poses a reduced risk for SEC intoxication through consumption of that food. In contrast to this, the *sed*-containing isolate Sa14-003 produced detectable SED after 72 h in both milk and meat, suggesting that although 16 °C poses a risk for SED intoxication, it requires prolonged temperature abuse to achieve this. Taken together, these results support the assertion that maintaining the cold chain is an effective control to prevent SEC or SED intoxication [24,32].

Production of enterotoxin at 30 °C, however, highlights a number of variations when comparing production in food matrices or by different isolates. Consistent with previous results, the milk matrix appeared to elicit lower levels of SEC production relative to other matrices, in this case MBB [23]. In contrast to this, the medium did not appear to significantly alter SED production by Sa14-003. It should be noted, however, that this may not be the case for every strain of *S. aureus* harbouring *sed*, with a previous study suggesting that, with some exceptions, the majority of strains do in fact produce less SED in milk relative to MBB [26].

When comparing the behaviour of *sec*-containing isolates to each other in a food matrix, significant differences were observed. When this was considered with respect to the phylogenetic analysis, a number of correlations were evident. Sa14-004, a bovine isolate which clustered together with other bovine isolates, produced the least amount of SEC when grown in MBB. The caprine (Sa14-006 and Sa14-007) and ovine (Sa13-004 and Sa13-005) isolates, on the other hand, produced significantly higher SEC relative to Sa14-004; the exception to this was the caprine isolate Sa13-003. Interestingly, this was the isolate with the frameshift mutation in a poly(A) homopolymeric tract of its *sec* gene. Such mutations are known to have a role in tightening gene regulation, enabling phase switching and inactivation, and may be influencing the transcription of *sec* in this isolate [52]. Further studies could help elucidate the role of this class of mutation in *S. aureus* enterotoxin production, particularly as such a mutation was also observed in the *sel* gene of other isolates in this study (Sa14-004 and Tokyo12381) suggesting this to be a wider feature of *S. aureus* enterotoxin gene regulation. Nonetheless, although genetically-related *sec* genes showed similar patterns in SEC production, in the case of the caprine/ovine genotype Sa13-004 produced a significantly higher concentration of SEC relative to other isolates in this genotypic subgroup, suggesting other factors are influencing gene expression and/or protein expression of this enterotoxin.

### 3.4. Phylogenetic Analysis of S. aureus Enterotoxin Genes sec and sel

Host adaptation of *S. aureus* clones has been identified for a number of clonal complex (CC) or sequence type (ST) subgroups, including CC97 and ST8 in cattle, CC133, CC522, and CC700 among ovine and/or caprine species, and the ST5 poultry-adapted clade [32,53,54,55]. Such trends in genetic evolution with host adaptation was observed with enterotoxin gene nucleotide sequences for both *sec* and *sel* in this study. Clustering of animal isolates distinct from human isolates when examining the *sec* sequence highlights this characteristic of the *S. aureus* population structure. Further segregation of bovine isolates from caprine/ovine isolates was also evident. Similar host-associated segregation was noted when examining the *sel* phylogeny. The bovine isolate in this study was ST8, whereas the caprine and ovine isolates were CC133, again highlighting the host-adapted associations of these subgroups.

## 4. Conclusions

This study provides insights into the growth and associated toxin production of two important enterotoxigenic bacterial groups, *B. cereus s. l.* and *S. aureus*. It highlights the high variability in behaviour when comparing strains, but also the dramatic effect food matrices exert on this. Of particular note was the behaviour of individual *B. cereus s. l* when grown in different food matrices, with marked differences in enterotoxin production when comparing milk to MBB. This highlights the difficulty in applying food safety legislation directed to the prevention of foodborne illness, as clearly criteria for a single food type may not be applicable to another. In addition, results of this study suggest certain strains appear to produce high amounts of diarrheal toxins even at what would be considered low contamination levels, with a contamination limit of 10^5^ not suitable to ensure safety in some cases. Taken together, these results suggest more detailed understanding of the behaviour of enterotoxigenic behaviour in food matrices is required to inform food safety risk assessment and form the basis of policy.

## 5. Materials and Methods

### 5.1. Isolates Included in This Study

A list of isolates included in this study are shown in Table S1. Seven isolates were used in this study for growth curve and enterotoxin analysis: for *B. cereus s. l.* these were Bc13-006 (*B. cereus s. s.*), Bc14-005 (*B. cereus s. s.*), Bc14-009 (*B. mycoides*), Bc14-011 (*B. mycoides*), Bc14-027 (*B. weihenstephanensis*), Bc14-026 (*B. weihenstephanensis*), and Bc13-016 (*B. thuringiensis*); for *S. aureus* these were Sa13-003, Sa13-004, Sa13-005, Sa14-003, Sa14-004, Sa14-006, and Sa14-007.

### 5.2. Preparation of Milk and MBB

For milk experiments, 10% reconstituted skim milk (Oxoid, Basingstoke, UK) was used/prepared as per the manufacturer’s instruction. MBB was prepared as previously described [56]. Briefly, diced bovine meat steak was boiled in water (1:2 ratio) for 20 min. The broth was then filtered through a coarse filter paper (Postlip ‘Crinkled Grey’ filter paper; Postlip, East Walpole, MA), and then vacuum filtered using Whatman No. 1 filter paper before being autoclaved (121 °C for 35 min). Sterile media was then dispensed into 50 mL aliquots, stored at −20 °C until required, and then thawed as needed. Analysis of the macronutrient and pH profiles of both broths are shown in Table 1.

### 5.3. Enterotoxin Gene Detection

Primers used for detection of enterotoxin genes in *B. cereus s. l.* isolates are listed in Table 2. Five genes were targeted: *nheA*, *hblA*, *bceT*, *hlyII*, and *entFM.* Amplification using primers from other studies (i.e., *bceT*, *hlyII*, and *entFM*) were as detailed in the associated reference listed in Table 2 [18,41,57]. For the primers designed in this study (i.e., *nheA* and *hblA*), the PCR conditions used were: one cycle of 95 °C for 15 min, followed by 35 cycles of 95 °C for 45 s, 57 °C for 60 s, and 72 °C for 60 s. The final concentrations of *nheA* or *hblA* primers was 25 nM. A final extension cycle of 72 °C for 10 min was used. All PCR reactions were carried out using HotStar Taq DNA polymerase and Master Mix (Qiagen, Hilen, Germany). All PCR reactions were carried out using SimpliAmp™ Thermal Cycler (Applied Biosystems, Foster City, CA, USA). Categorical differences clustering was performed using BioNumerics (v7.6.2) software (Applied Maths, Sint-Martens-Latem, Belguim).

### 5.4. Growth Curves

Fresh overnight cultures for each isolate were inoculated into the relevant test medium (i.e., either MBB or 10% reconstituted skim milk) and incubated at 30 °C (*B. cereus s. l.*) or 37 °C (*S. aureus*). For each growth curve growth, an initial inoculum level of approximately 10^3^ CFU/mL was used, by serially diluting overnight cultures in the test medium and inoculating to approximately 10^3^ CFU/mL initial cell concentration. For 16 °C growth curves, enumeration of cell numbers was performed at the following timepoints: 0 h, 6 h, 24 h, 30 h, 48 h, and 72 h, by preparing serial dilutions and using the direct plate count method on brain heart infusion agar. Samples for toxin detection were collected at 24 h, 30 h, 48 h, and 72 h time points, and processed as outlined below. For 30 °C growth curves, enumeration of cell numbers was performed at 0 h, 6 h, and 24 h time points, as described above. Samples for toxin detection were collected at 6 h and 24 h. All growth curve measurements were duplicated and included two biological replicates.

### 5.5. Enterotoxin Detection

For *B. cereus s. l.* toxin detection, the GLISA Duopath^®^ Cereus Enterotoxins kit (Merck, Darmstadt, Germany) was used, as per manufacturer’s instruction. Briefly, at each time point tested, 150 µL of sample was applied directly to the lateral flow device, and results were recorded after 20 min. A semi-quantitative scoring scheme was used based on the intensity of the reaction band as follows: a test with clearly no line was ‘-’ (i.e., negative), a test with a faint line was ‘+’, a test with a line as strong as the control line was ‘+++’, and a band with an intensity between ‘+’ and ‘+++’ was designated ‘++’. 

For *S. aureus* enterotoxin detection, the RIDASCREEN SET A, B, C, D, E (Enzyme Immunoassay for identification of *Staphylococcus* enterotoxins A, B, C, D, and E in food and bacterial cultures; R-Biopharm AG, Darmstadt, Germany) was used, according to manufacturer’s instructions. Briefly, 1 mL sample was centrifuged at 3500× *g* at 10 °C for 10 min, and 100 µL of the middle layer was then added to wells A–G and incubated at 37 °C for 1 h. Wells were then washed five times with 300 µL of wash buffer, 100 µL of conjugate 1 was added, and the wells were incubated for another hour at 37 °C. The wash step was repeated and 100 µL of conjugate 2 was added to the wells which were incubated for 30 min at 37 °C. The wash step was again repeated, 100 µL of the substrate/chromogen was added and the wells were incubated for 15 min at 37 °C at which point 100 µL of stop solution was added. Absorbance was read immediately using Varioskan^®^ Flash (Thermo Scientific, Waltham, MA, USA) with SkanIt™ software (Thermo Scientific) at 450 nm and 630 nm. All ELISA enterotoxin testing was performed on two biological replicates.

### 5.6. Phylogenetic Analysis of Toxin Genes

The *sec* gene sequences were extracted from de novo assemblies of the six isolates capable of producing SEC used in the growth curves and enterotoxin production tests of this study. An additional panel of *sec* gene sequences was compiled using publically available sequences from the NCBI database. All sequences were imported into the Geneious (v9.0.5) software platform [58]. Nucleotide alignments of representative unique sequences were performed using the translation alignment algorithm (global alignment with Blosum62 cost matrix), and then clustered with the PhyML algorithm, using the Hasegawa-Kishino-Yano substitution method with 1000 bootstrap replicates [59,60].

### 5.7. Statistical Analyses

Student *t*-tests were performed on each *S. aureus* isolate comparing results of enterotoxin production between the two food matrices and a one-way analysis of variance (ANOVA) was performed on each 30 °C *S. aureus* enterotoxin production dataset for each medium. When the differences across the group were significant (*p* < 0.01), a Tukey post-hoc test was performed.

## Figures and Tables

**Table 1:** Macronutrient analysis and characteristics of food matrices.

Medium	Protein	Fat	Carbohydrate	Moisture	Ash	pH
10% RSM	3.4%	0.15%	ND *	90%	0.8%	6.4
Meat-based broth	1.1%	0.1%	<0.1%	98.5%	0.4%	5.94

* ND, not determined. Milk carbohydrate is expected to be approximately 5%.

**Table 2:** Primers used in this study.

Primer Name	Gene Target	Annealing Temperature	Product Size	Reference
hblA-F	*hblA*	GTGGTGGATTGGGAGCAG	390 bp	This study
hblA-R		CTTGCATAGARTCGATATTATC		
nheA-F	*nheA*	TACAGGGTTATTGGTTACAGC	482 bp	This study
nheA-R		CACAATATCTCCACTTGATCCT		
bceT-F	*bceT*	GCTACGCAAAAACCGAGTGGTG	679 bp	Kim et al. (2015)
bceT-R		AATGCTCCGGACTATGCTGACG		
ENTA	*entFM*	ATGAAAAAAGTAATTTGCAGG	1269 bp	Asano et al. (1997)
ENTB		TTAGTATGCTTTTGTGTAACC		
Fhly-II	*hlyII*	GATTCTAAAGGMACTGTAG	868 bp	Adapted from Fagerlund et al. (2004)
Rhly-II		GGTTATCAAGAGTAACTTG		

## Supplementary Materials

The following are available online at www.mdpi.com/2072-6651/9/7/225/s1, **Table S1.** Isolates included in this study.
