# Supplementary material for: Characterization of Enterotoxigenic Bacillus cereus sensu lato and Staphylococcus aureus Isolates and Associated Enterotoxin Production Dynamics in Milk or Meat-Based Broth
Source: Toxins (Basel). 2017 Jul 15;9(7):225. doi: 10.3390/toxins9070225 (PMC5535172; doi:10.3390/toxins9070225)
Supplement: Supplementary file 1 [file toxins-09-00225-s001.pdf]

Supplementary Materials: Characterization of Enterotoxigenic *Bacillus cereus sensu lato* and *Staphylococcus aureus* Isolates and Associated Enterotoxin Production Dynamics in Milk or Meat-Based Broth

Laura Walker-York-Moore, Sean C. Moore and Edward M. Fox

Table S1. Isolates included in this study.

Isolate	Species	Source	Country	Genbank Accession Number
This study				
Bc13-004	*B. thuringiensis*	Soil	Australia	Not sequenced
Bc13-006	*B. cereus s. s.*	Bovine faeces	Australia	Not sequenced
Bc13-014	*B. thuringiensis*	Caprine faeces	Australia	Not sequenced
Bc13-016	*B. thuringiensis*	Caprine faeces	Australia	Not sequenced
Bc14-001	*B. cereus s. s.*	Bovine faeces	Australia	Not sequenced
Bc14-005	*B. cereus s. s.*	Milk filter	Australia	Not sequenced
Bc14-006	*B. pseudomycoides*	Milk filter	Australia	Not sequenced
Bc14-009	*B. mycoides*	Soil	Australia	Not sequenced
Bc14-011	*B. mycoides*	Bovine faeces	Australia	Not sequenced
Bc14-015	*B. cereus*	Caprine faeces	Australia	Not sequenced
Bc14-026	*B. weihenstephanensis*	Animal feed	Australia	Not sequenced
Bc14-027	*B. weihenstephanensis*	Milk filter	Australia	Not sequenced
Sa13-003	*S. aureus*	Raw milk	Australia	MAQK00000000
Sa13-004	*S. aureus*	Raw milk	Australia	MAQL00000000
Sa13-005	*S. aureus*	Milk filter	Australia	MAQM00000000
Sa14-003	*S. aureus*	Milk filter	Australia	MAQQ00000000
Sa14-004	*S. aureus*	Raw milk	Australia	MAQR00000000
Sa14-006	*S. aureus*	Raw milk	Australia	MAQT00000000
Sa14-007	*S. aureus*	Raw milk	Australia	MAQU00000000
Others				
ED133	*S. aureus*	Ovine	France	CP001996.1
FDAARGOS_159	*S. aureus*	Human	USA	CP014064.1
FORC_027	*S. aureus*	Human	South Korea	CP012692.1
FRI-913	*S. aureus*	Prawns (food)	England	X51661.1
H-EMRSA-15	*S. aureus*	Human	Unknown	CP007659.1
N315	*S. aureus*	Human	Japan	BA000018.3
NCCP14558	*S. aureus*	Human	Korea	CP013953.1
NCCP14562	*S. aureus*	Human	South Korea	CP013955.1
NN50	*S. aureus*	Human	Japan	AB679717.1
NZAK3	*S. aureus*	Human	New Zealand	LT009690.1
RF122	*S. aureus*	Bovine	Ireland	AJ938182.1
SA564	*S. aureus*	Human	Switzerland	CP010890.1
SAI3	*S. aureus*	Human	Switzerland	KX168614.1
SAI48	*S. aureus*	Human	Switzerland	KX168615.1
SaPIbov	*S. aureus*	Bovine	Ireland	AF217235.1
SAR1	*S. aureus*	Bovine mastitis milk	Switzerland	KX168613.1
Tokyo12381	*S. aureus*	Human	Japan	AB860418.1
Tokyo12571	*S. aureus*	Human	Japan	AB860417.1
ZJ5499	*S. aureus*	Human	China	CP011685.1
